# A systematic review including meta-analysis of work environment and depressive symptoms

**DOI:** 10.1186/s12889-015-1954-4

**Published:** 2015-08-01

**Authors:** Töres Theorell, Anne Hammarström, Gunnar Aronsson, Lil Träskman Bendz, Tom Grape, Christer Hogstedt, Ina Marteinsdottir, Ingmar Skoog, Charlotte Hall

**Affiliations:** Stress Research Institute, Stockholm University, SE-106 91 Stockholm, Sweden; Department of Neuroscience, Karolinska Institutet, SE- 171 77 Stockholm, Sweden; Department of Public Health and Clinical Medicine, Department of social medicine, Umeå University Hospital, SE-901 85 Umeå, Sweden; Department of Psychology, Stockholm University, SE-106 91 Stockholm, Sweden; Division of Psychiatry, Lund University, SE-221 00 Lund, Sweden; South Health Care Centre, SE-761 46 Norrtälje, Sweden; Division of Occupational medicine, Institute for Environmental Medicine, Karolinska Institutet, SE-171 77 Stockholm, Sweden; Division of Psychiatry, Linköping University, SE-581 83 Linköping, Sweden; Department of Psychiatry and Neurochemistry, Göteborg University, SE-411 24 Stockholm, Sweden; Swedish Council of Health Technology Assessment, SE-103 59 Stockholm, Sweden

**Keywords:** Depression, Work stress, Prevention, Ergonomic, Toxicology

## Abstract

**Background:**

Depressive symptoms are potential outcomes of poorly functioning work environments. Such symptoms are frequent and cause considerable suffering for the employees as well as financial loss for the employers. Accordingly good prospective studies of psychosocial working conditions and depressive symptoms are valuable. Scientific reviews of such studies have pointed at methodological difficulties but still established a few job risk factors. Those reviews were published some years ago. There is need for an updated systematic review using the GRADE system. In addition, gender related questions have been insufficiently reviewed.

**Method:**

Inclusion criteria for the studies published 1990 to June 2013: 1. European and English speaking countries. 2. Quantified results describing the relationship between exposure (psychosocial or physical/chemical) and outcome (standardized questionnaire assessment of depressive symptoms or interview-based clinical depression). 3. Prospective or comparable case-control design with at least 100 participants. 4. Assessments of exposure (working conditions) and outcome at baseline and outcome (depressive symptoms) once again after follow-up 1-5 years later. 5. Adjustment for age and adjustment or stratification for gender.

Studies filling inclusion criteria were subjected to assessment of 1.) relevance and 2.) quality using predefined criteria. Systematic review of the evidence was made using the GRADE system. When applicable, meta-analysis of the magnitude of associations was made. Consistency of findings was examined for a number of possible confounders and publication bias was discussed.

**Results:**

Fifty-nine articles of high or medium high scientific quality were included. Moderately strong evidence (grade three out of four) was found for job strain (high psychological demands and low decision latitude), low decision latitude and bullying having significant impact on development of depressive symptoms. Limited evidence (grade two) was shown for psychological demands, effort reward imbalance, low support, unfavorable social climate, lack of work justice, conflicts, limited skill discretion, job insecurity and long working hours. There was no differential gender effect of adverse job conditions on depressive symptoms

**Conclusion:**

There is substantial empirical evidence that employees, both men and women, who report lack of decision latitude, job strain and bullying, will experience increasing depressive symptoms over time. These conditions are amenable to organizational interventions.

## Background

Depressive symptoms are potential outcomes of poorly functioning work environments. Such symptoms are frequent and may cause considerable suffering for the employees themselves as well as financial loss for the employers. Accordingly good prospective studies of psychosocial working conditions and depressive symptoms are valuable.

Several reviews including prospective studies of psychosocial factors at work in relation to depression have been published. Bonde [1] concluded that there were consistent findings that perception of adverse psychosocial factors in the workplace is related to an elevated risk of subsequent depressive symptoms or major depressive episode but also that methodological limitations preclude causal inference. Netterström et al. [2] made a similar conclusion but pointed out that studies are needed that assess in more detail the duration and intensity of exposure necessary for developing depression. The conclusions in a review by Siegrist from the same year [3] were similar. Also, Michie and Williams [4] concluded that” many of the work related variables associated with high levels of psychological ill health, are potentially amenable to change which has been shown in intervention studies that have successfully improved psychological health and reduced sickness absence”. A review of psychosocial and health effects of workplace reorganization by Egan et al. [5] concluded that” some organizational-level participation interventions may benefit employee health, as predicted by the demand-control model”. However, several other psychosocial exposures should be examined more in detail.

Most of the work environment reviews published so far have not been confined to depression only - they have included for instance stress related disorders, psychologically related sick leave and suicide or combinations [4, 6–8] as outcomes, and it has sometimes been difficult to disentangle them. Studied work environment factors have mostly been limited to psychosocial factors although two reviews have included physical/chemical/ergonomic exposures as well. The conclusion from them [4, 7] was that the evidence for physical/chemical/ergonomic exposures is limited and inconclusive. Nieuwenhuijsen et al. [8] published a review of the effects of the psychosocial environment on risk of stress-related disorders (SRDs) and concluded that there is” strong evidence that high job demands, low job control, low co-worker support, low supervisor support, low procedural justice and a high effort- reward imbalance predicted the incidence of SRDs”.

In summary, the evidence about the negative impact of certain work environments for depressive symptoms is accumulating but so far there has been no review taking the entire spectrum of adverse working conditions into account and at the same time focusing on depressive conditions/symptoms as outcome. Most of the reviews have used multiple kinds of mental health outcomes. However, depression is the most widely reported outcome in the field of mental health in epidemiological research. Depressive symptoms are well understood in psychiatry which has resulted in a large number of studies. Accordingly this outcome should provide a good basis for a focused systematic review. As far as the authors know there is no published study that has used the international GRADE system [9] for evaluating the evidence in this field. In addition there is a need for a systematic review utilizing the most recent developments in search technology.

An important aspect of the systematic review process is to systematically and transparently assess the scientific evidence. We have chosen to use the internationally recognized GRADE- system for scientific evaluation. The GRADE system uses four levels of evidence, namely High, Moderate, Limited and Very Limited. We are well aware that the system has been developed primarily for assessing interventions in a health care context, but the system has been adapted to epidemiological evaluation. Beside the transparency, an advantage is that the GRADE system [9] - a system often applied in reviews conducted within the Cochrane Collaboration - is increasingly used internationally e.g., by the World Health Organization. Hence results from systematic reviews can be more easily compared.

Time has elapsed since most of the previous reviews were published and new studies are published continuously. The most relevant reviews were published in 2008. They pointed at several methodological shortcomings, and it is not known whether researchers more recently have tried to address the identified scientific problems. In particular, the reviews have pointed at the paucity of studies on physical/chemical/ergonomic exposures.

A topic that has not been addressed sufficiently in previous reviews is gender in the relationship between working conditions and the development of depressive symptoms. Are the associations different for men and women?

### Aim of the study

The aim of this study was to provide systematically graded evidence for possible associations between work environment factors and near-future development of depressive symptoms

## Methods

The present review was based upon studies with a prospective design and is focused on the relationship between working conditions and development of symptoms of depression among the employees.. We conducted and funded this systematic review within the framework for the Swedish Council on Health and Technology Assessment, a public agency with the charge of providing impartial and scientifically reliable information to decision makers and health care providers [10].

### Search strategy

Systematic literature search was performed in the following data bases: PubMed, Embase, Psycinfo, Arbline (Swedish database), Cochrane library and NIOSHTIC-2. A combination of controlled search words (e.g., MeSH) and free- text words was used. The search strategy for the outcome was performed for mesh terms (‘Depression’ and ‘Depressive Disorders’) and as free search in title and abstract (depress* and dysthym*). The whole search strategy is available at http://www.sbu.se/upload/Publikationer/Content0/1/223E/Inclusion%20criteria_occupational%20exposure_depression_burnout.pdf. We only accepted as articles in scientific journals with independent reviews.

### Inclusion criteria

The inclusion criteria for studies were:

1. The study should have examined the importance of the work environment for depressive symptoms. Our review was not confined to any specific kind of work environment factors. Physical/chemical/ergonomic exposures as well as psychosocial factors were screened.
2. The study should be relevant for Swedish conditions and focused on people at work. Work environments in Europe, North America, Australia and New Zealand were included.
3. In the study symptoms of depression should have been analyzed. These should have have been certified through diagnostic investigation or with established scales. We argued that not only diagnosed major depression, but also milder states with depressive symptoms are relevant since depressive feelings give rise to suffering, increase the risk of long term sick leave and cause productivity decline and quality loss in work places [11]. Thus, our review included both studies with standardized clinical interviews regarding diagnosed depression and studies based upon rating scales on depressive symptoms. As diagnosed depression is also to a large extent based on symptoms we decided that the most accurate naming of the outcome of our review was depressive symptoms. A few studies were based upon either sick leave data or registered anti-depression medication as outcome but these studies are not included in this review.
4. A minimum of 100 persons should have been included in the exposed group and the results were controlled for at least age and gender.
5. The study should have been published between the years 1990 and (June) 2013 and written in English.
6. Prospective or comparable case-control design. Only prospective cohort, case control (with design equivalent to prospective) and randomized intervention studies with at least 100 participants were included. By case control studies with “design equivalent to prospective” we are referring to studies with strict definition of cases recruited in a representative way in the same population as the control group.

Assessments of exposure should have been made before disease onset.

Doublets were systematically identified and only the most relevant publication in a doublet was included.

### Analyses of relevance and quality

Abstract screening and full-text assessment were conducted by a specialist in occupational medicine and a psychiatrist.

After that, the scientific experts started their examination. Pre-set evaluation forms were used. The experts judged relevance and quality of the studies on the basis of the relevance/quality criteria, their experience as researchers and their knowledge of the field. Accordingly they were recruited among Swedish academic high ranking specialists in fields of relevance for the process, namely psychiatry (three), epidemiology and stress research (three), work psychology (one) and family practice (one). This group was divided into pairs with as widely differing specialty in the pair as possible. In the following process, the articles remaining in the process were randomly assigned to the four pairs (with avoidance of author bias). Concordance in judgments of relevance and quality was trained. After the training session, each member of the pair did the assessments separately, and then discordances were discussed within the pair. If disagreement remained another pair was asked to make an independent judgment. If that decision was in disagreement with the first group, we made the decision in the whole group.

In the first expert phase, the group judged relevance. Relevance criteria are presented in http://www.sbu.se/upload/Publikationer/Content0/1/223E/Inclusion%20criteria_occupational%20exposure_depression_burnout.pdf.

Secondly, we performed a quality assessment. Three levels of quality rating were used, (low, medium high and high quality) and in the final grading process only those with medium high and high quality were accepted. Accordingly the important dividing line was between poor and medium high quality whereas the distinction between medium high and high was less crucial. Studies on the borderline between low and medium high quality were accordingly re-examined by the whole group. A list of relevant articles meeting the inclusion criteria judged to be of low quality is available at http://www.sbu.se/upload/Publikationer/Content0/1/223E/Inclusion%20criteria_occupational%20exposure_depression_burnout.pdf

The following aspects of quality were considered:

1. Representativeness of study sample. Representativeness and ways of defining and recruiting the sample as well as attrition in different steps were considered in the quality rating. Statistical considerations and an insightful discussion of possible consequences of a possible systematic drop-out for findings were required in case of marked drop-out problems.
2. Confounding. Age and at least some aspect of socioeconomic conditions should have been considered. Gender specific analyses were preferred but when such analyses were not available, adjustment for gender was required. Life habits such as smoking habits and alcohol consumption were not taken into account as confounders in our review.
3. Prospective data collection. All results of the studies included in this review (apart from case-control studies) are based upon assessments of exposure and depressive symptoms in the beginning and of the depressive symptoms again at least one year later. In the calculations of associations a design with either exclusion of subjects with depressive symptoms at baseline or adjustment for baseline level of depressive symptoms was required. Qualified statistics and thorough discussion of longitudinal data rendered higher quality ratings.
4. For both exposure and outcome assessment, psychometrically standardized and validated methods were required. Well established methods enable comparison across studies and therefore contributed to higher quality rating.
5. Designs that enable the analysis of a dose response relationship contributed to a high quality rating. For instance, in a few studies the work environment was assessed in two or three subsequent waves and the development of depressive symptoms followed up after the last assessment. Exposure to given work environment factor on one, two or three occasions could be regarded as a progressive duration of exposure and was regarded as equivalent of a dose-response analysis.

Even between studies of specific work environment factors there were differences with regard to operationalization of exposure. Examples are job strain (combination of high psychological demands and low decision latitude) and effort reward imbalance (combination of high effort and poor reward). Since the overall aim of the present study was to grade total evidence, not to assess magnitude of associations, and since it was impossible to re-construct operationalizations in such a way that they would match one another we decided to use the definitions presented by the authors themselves and to mostly abstain from assessment of overall magnitude of the different relationships.

The final list of studies judged to be of high or medium high quality is listed in Appendix.

### GRADE procedure

An important aspect of the systematic review process was to systematically and transparently assess the scientific evidence. According to the GRADE instructions explicit consideration should be given to each of the GRADE criteria for assessing the quality of evidence (risk of bias/study limitations, directness, consistency of results, precision, publication bias, magnitude of the effect, dose-response gradient, influence of residual plausible confounding and bias “antagonistic bias”) although different terminology may be used. For level 4 (=High), randomized trials are required and there were no such published relevant studies in our search. For observational studies of the kind included in the present review, the highest possible grade is Moderate = 3 if there is sufficient reason for an upgrading from the normal level for such studies of 2 (=Limited). Level 1 (=Very limited) corresponds to evidence based on case reports and case series or on reports downgraded evidence from observational studies.

We allowed for upgrading the scientific evidence when there was strong coherence of results between studies - according to the most recent guidelines [12]. Accordingly when there were many published observational studies of medium high or high quality with homogenous results (almost all pointing in the same direction although all findings may not have been statistically significant) the evidence was graded on level 3 (two exposures, high decision latitude as protective and job strain as negative exposure, see below). Level 3 can also be used according to the GRADE system even when there are relatively few studies if there are unanimous findings with high odds ratios (above 2.0). This occurred for one exposure – bullying (see below).

### Meta-analyses/Forest plots

In the studies results were reported as calculations of association, e.g., expressed as odds ratios, from multiple logistic regression, multivariate correlations or multiple linear regression coefficients. Whenever possible, the results were transformed into multiple logistic regression odds ratios. Forest plots were used for visual interpretation. To assist in illustrating the results, and as a contribution to the overall assessment, these forest plots (meta-analyses) were conducted when in at least two studies the same risk factor was analysed and mathematically comparable data was provided using the Comprehensive Meta-Analysis software package (www.meta-analysis.com/index.php). Since the participants in the various studies might be construed as coming from the same population (workers) or from different populations (i.e., according to each study’s inclusion criteria) we chose to use a fixed effects model. The strength of the scientific evidence, using data from all of the included studies (not just those illustrated in the meta-analyses), was determined by pairs of the authors of this paper and then discussed and confirmed by all authors. Informal homogeneity tests were performed in order to compare results from studies using standardized depression interviews versus self-reported questionnaires, high quality versus medium high quality studies, general population studies versus specific occupational cohorts and men versus women. In these tests, we conducted sub-analyses of the presented findings and compared results between the sub-categories, e.g., if the association between job exposure and depressive symptoms differed according to the instrument used for assessing the symptoms.

### Ethics

All studies perused in this review have been approved by the scientific ethical committees in their universities. They have all been published in international scientific journals with peer review. Accordingly, no additional ethical approval has been required.

## Results

**Fig. 1:**
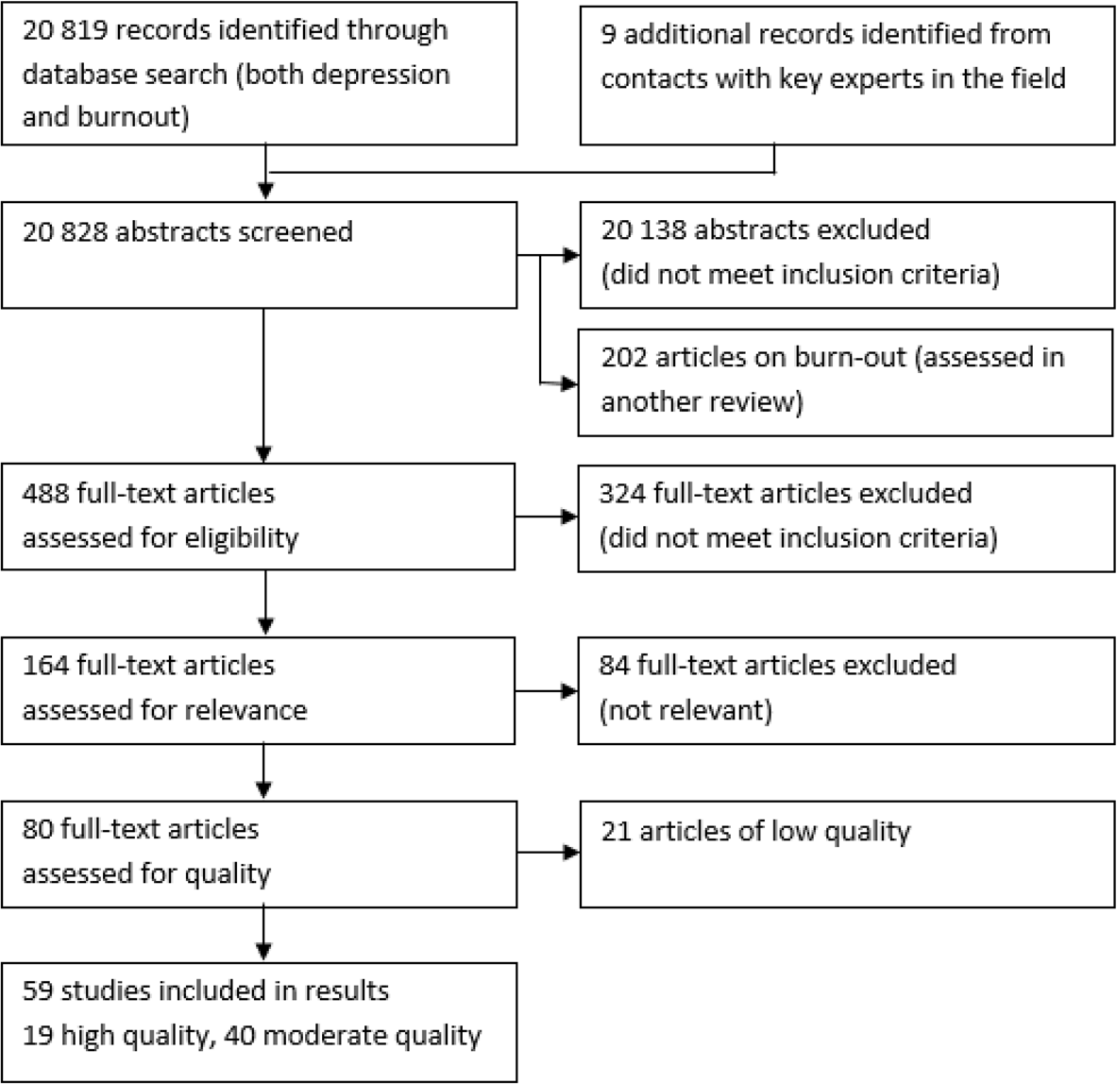
Flow chart of the literature search, screening, review- and quality assessment

Figure 1 shows the number of articles that were perused in the different steps. The process also included burnout as outcome. The results of the burnout review will be reported elsewhere. Altogether 20 828 articles were screened in the initial search process, and 488 of those were eligible in the review of depressive symptoms (and 202 for the review of burnout). 324 full text articles with depression as outcome were found not to fill inclusion criteria. Hence, 164 studies remained for relevance assessment. 84 of those were judged as not relevant and hence 80 studies were assessed with regard to quality. 19 were judged to be of high, 40 of moderately high and 21 of low quality. The grading of evidence has been based upon the 59 relevant studies with high/medium high quality. A detailed table showing the full results of the data extraction is available at http://www.sbu.se/upload/Publikationer/Content0/1/223E/Inclusion%20criteria_occupational%20exposure_depression_burnout.pdf.

Most studies were based on population samples although studies of samples from companies and occupational groups were also present. Few studies that were judged to be relevant were based upon objective assessments of exposure. Subjective assessments based upon standardized and validated questionnaires (for instance demand/control/support, effort/reward, procedural justice and bullying) were used in most studies. The most widely used established questionnaires rendered high quality ratings. With regard to depression outcome, both standardized interviews (mostly Composite International Diagnostic Interview, CIDI) performed by trained interviewers and different versions of standardized questionnaires (such as Center for Epidemiological Studies- Depression Scale, CES-D, and Hospital Anxiety and Depression Scale, HAD, and Hamilton Depression Scale, HRSD) for depressive symptoms were used.

Table 1 shows the results of the evidence grading process. Three exposures, two harmful (job strain and bullying) and one protective (control/decision latitude) were judged to have moderate evidence (grade 3) while 18 exposures were judged to have limited (grade 2) evidence. Ten exposures were judged to have very limited evidence (grade 1). Three of the exposures judged to have very limited evidence were related to heavy metals and other chemical exposures. The most extensively studied factors were decision latitude (158 251 subjects in 19 studies) and job strain - the combination of high psychological demands and low decision latitude (197 682 subjects in 14 studies). It was possible to compute a weighted odds ratio 1.74 (95 % CI 1.54 to 1.96 for studies with odds ratio calculations). A high decision latitude protected statistically against worsening depressive symptoms – with a weighted odds ratio of 0.73 (95 % CI 0.68 to 0.77). Bullying had been studied in 15 173 subjects in three studies. One of these studies showed results for men and women separately. Despite the relatively small number of studies, bullying was judged to be related to worsening depressive symptoms with an evidence grade of 3 as the findings were very consistent and the odds ratios were high (the weighted odds ratio being 2.82; 95 % CI 2.21 to 3.59).

**Table 1:** A summary of the scientific evidence for variables with sufficient data to draw a conclusion on the association between work environment factors and future depressive symptoms

Work-related factor	Participants	Studies	Scientific evidence
*Relationship between occupational environment and less depressive symptoms*			
Control	158 251	19	
*Relationship between occupational environment and more depressive symptoms*			
Demands - psychological job demands	53 985	10	
Job strain	197 682	14	
Passive job (low decision latitude, low job demands)	11 419	2	
High pressure job	34 554	5	
Effort reward imbalance	27 136	3	
Low support at the work place	82 772	17	
- Low supervisor support	50 935	8	
- Low co-worker support	27 170	6	
Poor social climate at the work place	9 242	2	
Poor social capital at the work place	59 340	2	
Low work place justice	33 589	5	
- Procedural injustice	33 589	5	
- Relational injustice	30 761	3	
Work place conflicts	13 732	3	
- Conflicts with superiors	9 692	2	
- Conflicts with co-workers	9 692	2	
Bullying	15 173	3	
Low job development	15 173	4	
Job insecurity	24 833	7	
Long working week	13 107	6	
*The scientific evidence is in-sufficient* () *to determine if there is a relationship between the following occupational factors and depressive symptoms*/			
Demands (several types of demands), Demands (emotional), Distributive justice, Threats, Violence, Irregular, Irregular work hours, Physically demanding work, Pesticides, Solvents, Heavy metals			

- There is scientific evidence for an association between exposure and outcome. The result is based on studies of high or moderate quality. The quality of evidence has been upgraded due to consistency of the data (control and job strain) or large magnitude of effect (bullying)

- There is scientific evidence for an association between exposure and outcome. The result is based on studies of high or moderate quality

- It is not possible to determine if there is any association between exposure and outcome. The motivation is that one or several conditions apply: 1) no study fulfilled the inclusion criteria, 2) none of the studies fulfilling the inclusion criteria were relevant to the hypothesis tested in the present review, 3) all relevant studies were of low quality or 4) studies were of high or moderate quality - but one or several limitations applied, e.g. inconsistency of data between studies

**Fig. 2:**
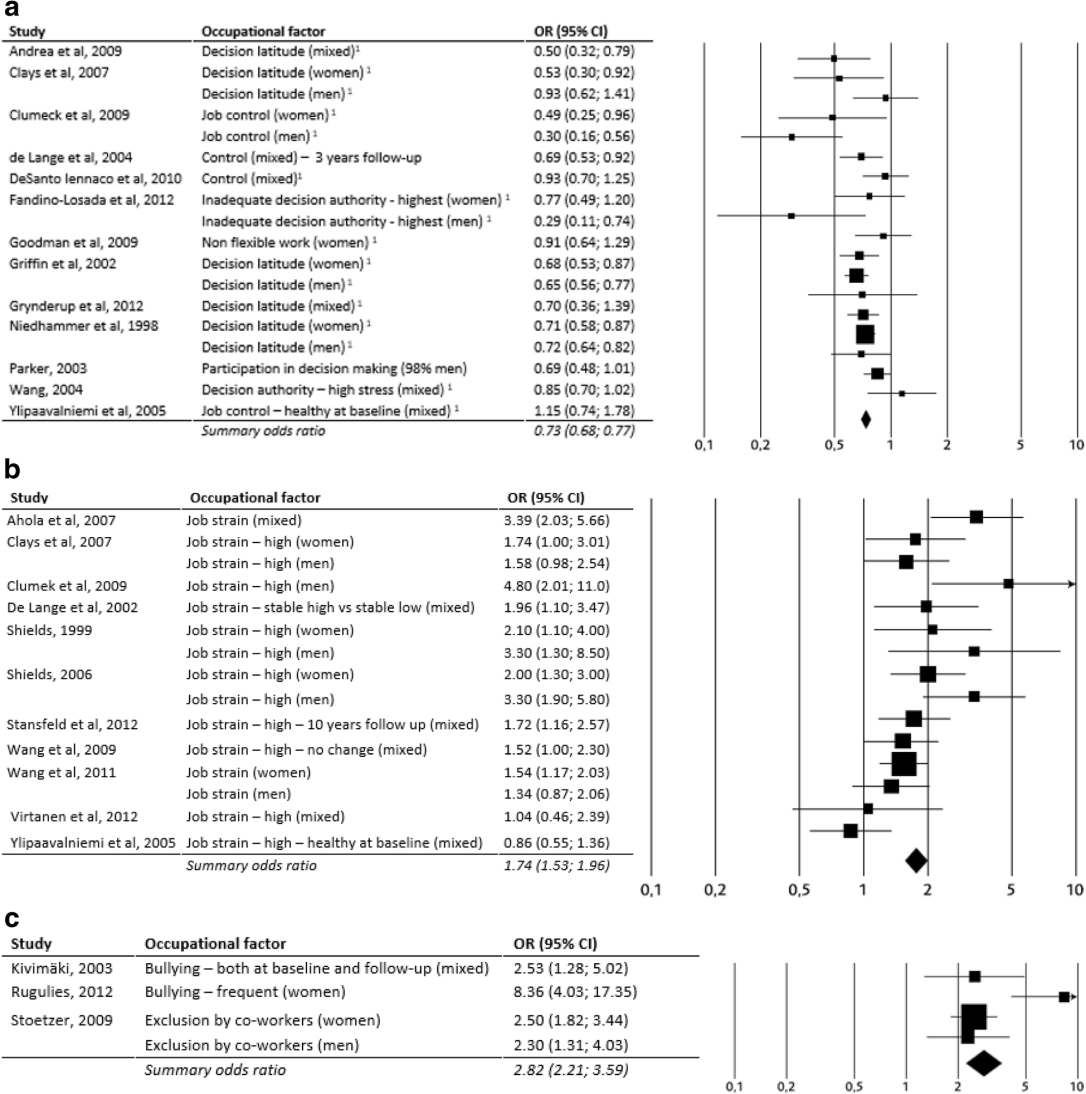
Association between work environment factors and development of depressive symptoms when evidence was judged as moderate (grade 3), **a**. Decision latitude, The graph is based on data from the least adjusted model in studies expressing the strength of the association either as odds ratios or as correlations (the latter have been transformed into odds ratios). Ylipaavalniemi et al.: “Healthy at baseline” refers to a doctor diagnosis/non-diagnosis of depression. Please note that data from six more studies (Dagher et al. 2011, Magnusson Hansson et al. 2009, Paterniti et al. 2002, Plaisier et al. 2007, Rugulies et al. 2006 and Wieclaw et al. 2008) are included in the evidence-rated result; however data from these studies could not be illustrated in the graph due to the data format. Data have been re-calculated to show the association between high level of control and development of depressive symptoms (data in these studies are presented as association between low level of control and depressive symptoms). **b**. Job strain, The graph is based on data from the least adjusted model in studies expressing the strength of the association either as odds ratios or as correlations (the latter have been transformed into odds ratios). Please note that data from three more studies (Ibrahim et al. 2009, Wieclaw et al. 2008 and Mantyniemi et al. 2012) are included in the evidence-rated result; however data from these studies could not be illustrated in the graph due to the data format. **c**. Bullying, The graph is based on data from the least adjusted model in studies expressing the strength of the association either as odds ratios or as correlations (the latter have been transformed into odds ratios)

Figure 2 shows forest plots for the three factors with evidence grade 3 - decision latitude (a), job strain (b) and bullying (c). For high decision latitude, 17/18 point estimates were lower than 1.0 (separate point estimates for men and women in five studies). The upper 95 % confidence limit was above 1.0 in five studies. For job strain, 14/15 point estimates were above 1.0. Three lower confidence limits reached below 1.0. The forest plots were based upon studies from which odds ratios could be extracted or calculated. It should be pointed out, however, that the total evidence grading also included a few additional studies. Bullying, finally, had four point estimates in the diagram. All of those were higher than 2.0 and all the lower confidence limits were above 1.0.

The exposures with a limited level of evidence were psychological demands (quantitative psychological demands defined according to the widely used Job Content Questionnaire or alternative psychometrically tested versions), the combination of low psychological demands and low decision latitude (“passive work”), “pressing work” (mainly important life events at work), effort reward imbalance, low social support (from management and coworkers), poor social climate, poor social capital, low procedural and relational justice, conflicts with superiors and colleagues, poor skill discretion, job insecurity and long working weeks (the latter for women only).

The exposures with very limited (= level 1) evidence were other kinds of demands (not quantitative) including emotional demands, distributive justice, threats, violence, irregular working hours, long working hours (men), physically demanding work, exposure to pesticides and insecticides, solvents and heavy metals.

Homogeneity tests showed that results were comparable for two groups of outcome measures (standardized interview versus standardized self-report questionnaire), for men and women, for general population versus specific occupation cohorts and for white collar versus blue collar groups.

## Discussion

### Main findings and recent developments in the field

The aim of the study was to provide systematically graded evidence for possible associations between work environment factors and near-future development of depressive symptoms. A total of fifty-nine relevant articles with high or medium high scientific quality fulfilling our criteria were found. The results provide evidence for several work conditions being linked to depressive symptoms among the employees in both positive and negative directions. Scientific evidence of grade three out of four (in other words moderately strong) was shown for job strain (high psychological demands and low decision latitude), low decision latitude and bullying. Furthermore, scientific evidence of grade two was found for psychological demands, effort reward imbalance, low support, unfavorable social climate, lack of procedural and relational justice, conflicts with superiors and colleagues, limited skill discretion, job insecurity and long working week.

An important finding is that there were few prospective studies with sufficient quality of the relationship between adverse chemical (pesticides and heavy metals for instance) and physical (heavy loads, awkward positions, irradiation, cold and hot temperature) and depressive symptoms. This field needs more research.

The results should primarily be interpreted in the context of the Western world. We deliberately limited our inclusion of studies to these countries. The rationale behind this was that we wanted to secure similar cultural framework around work in order to simplify our interpretation of the findings.

The review differs from earlier studies in the field due to its comprehensive and thorough approach. Our review is based on an extremely thorough literature search as well as on a well-described and systematic evaluation of a large number of publications. Thus, it includes all kinds of environmental exposures, physical as well as psychosocial and that it is based upon a systematic approach. This is the first review in which the examination of evidence follows (a slight modification of) GRADE principles. Furthermore it is including more recently published research than previous reviews.

Our review shows that the psychosocial research field has made progress since the reviews published in 2008 and 2010. Bonde [1] and Netterström et al. [2] made critical remarks about possible publication bias, lack of more “objective” measures of exposure and outcome and also about lack of time perspectives which would be needed for the understanding of time of exposure needed for the development of depression. With regard to objective measures, there are more published studies than previously with standardized interview based assessment of clinical depression. Comparison of the plots corresponding to results from studies based upon standardized interviews did not differ from those from studies based upon internationally accepted depression questionnaires. Objective exposures are still uncommon, however. One interesting approach was used by Virtanen et al. [13] who could show that hospital staff who experienced excess occupancy of hospital beds had increased risk of developing sick leave because of depression in a dose-response manner, with excess occupancy exceeding 10 % being associated with an odds ratio of sick leave for depression of 1.94 (1.14-3.28).

During later years research designs on the association between work environment factors and depressive feelings have become increasingly sophisticated. For instance, Shields [14], Stansfeld et al. [15], De Lange et al. [16] and Wang et al. [17] have examined possible effects of exposure to job strain at least twice, or even three times in the follow-up survey waves. Their findings indicate that accumulated or increasing job strain has a stronger adverse statistical effect on risk of experiencing increased ratings of depressive symptoms during follow-up than decreasing job strain. As might be expected, these studies show that two or more assessments of the job situation provide more precise information regarding risk than only one measurement. Therefore stronger evidence regarding the influence of working conditions on mental health may be expected in future research with a growing body of studies with such methodology.

The literature search included articles published up to June 2013. For practical reasons it has not been possible to do a full review of the articles published after that date. However, a more informal search in the scientific literature (PubMed and PsycInfo until February 2015) showed that a few more recent prospective studies of work environment and development of depressive feelings relevant to the present review have been published. None of those would have changed our conclusions. Four of them support the use of standardized measures of job strain or high psychological demands and low decision latitude in predicting either depressive symptoms or major depressive disorder [18–21] and one of them supports the use of effort reward imbalance (or low reward) in the prediction of disability pension due to depression [21].

### Gender

Our results showed that similar work conditions were related to a similar increase in depressive symptoms among men and women. However, although there is no gender difference in excess risk associated with adverse work conditions, studies have shown that women actually have higher levels of job strain than men [22]. This may be one reason for women’s higher prevalence of depressive symptoms. Other studies indicate that work conditions can affect men and women differently in relation to development of major depressive disorder (MDD). For example, a Canadian study showed that men had elevated risk of MDD only if they were exposed to extremely high level of job strain while women had elevated risk of MDD even when exposed to moderate job strain [23]. The study points to the need of contextualizing findings about mental health and it may also illustrate that gender could be more relevant for the relationship between working conditions and major depressive disorder than for the relationship between working conditions and depressive symptoms.

### Technical issues

In this review we have not reviewed evidence whether there is interaction or not between high psychological demands and low decision latitude (as discussed for instance in Karasek and Theorell [24]). We have regarded the combination simply as a theoretical construction and evaluated its possible success or lack of success as a predictor of development of depressive symptoms.

In forest plots, we chose to use data from the least adjusted model from each study. The main rationale for this was that these models were more comparable between studies than other models, since the more adjusted ones were adjusted to widely different potential confounders. The most powerful prognostic factor for incident depressive symptoms was manifest symptoms at the study baseline; a parameter that had to be assessed in each of the included studies. Generally, adjusting for other confounders had very little effect. For transparency, we have listed data in both least and most adjusted models, see extensive tables at http://www.sbu.se/upload/Publikationer/Content0/1/223E/Inclusion%20criteria_occupational%20exposure_depression_burnout.pdf.

An important point is that if a study presented data in several statistical models, all data from all models were included in the expert group assessment of scientific evidence for all of the results presented in this systematic review.

Assessments of odds ratios may be somewhat unreliable due to differences in methodology across studies and also due to the fact that summary odds ratios could not be calculated for some of the occupational exposures. It should however be pointed out that for most of the studied exposures the observed risks were of moderate size.

The operationalization of job strain differed between the studies. The majority of the published studies used the median split definition (above median for the psychological demands score and below median for the decision latitude score). When exposure to job strain is defined in this way and the remaining participants in the study are defined as unexposed there is relatively little contrast between unexposed and exposed subjects. This may lead to underestimation of the true association.

As recommended in the epidemiological literature we produced funnel plots to investigate possible publication bias. When there is pronounced publication bias, studies reporting “confirming” odds ratios with wide confidence intervals are more common than studies reporting “rejecting” odds ratios with wide confidence intervals. Such an analysis cannot replace a real analysis of publication bias – the best analysis would be to contact researchers asking for unpublished studies. But according to our exploration of the material, there was no such evidence of publication bias.

### Limitations

Most studies were based upon self-reports of both working conditions and depressive symptoms. Few of the studies were based upon in situ investigation of the work environment and standardized clinical interviews of employees. Such interviews are more objective and may more often identify depression than standardized self-rating questionnaires which primarily have screening or follow-up indications. The risk of inflated associations may arise, when there are subjective descriptions both of explanatory and dependent factors [25]. This is particularly the case in cross-sectional studies while in prospective studies this risk is less pronounced. The risk of inflated association decreases as adjustments are performed for initial symptoms of mental disease and when the assessments of working conditions and mental symptoms are standardized. Accordingly, in this study we only included prospective studies (and comparable case-control studies) with data on initial symptoms and standardized measures of exposure and outcome.

Due to the fact that the researchers in the included studies had chosen a wide range of different statistical measures to express associations between occupational exposure and depressive symptoms, it was not possible to conduct formal mathematical homogeneity analyses including the entire data material. Instead, the expert group conducted a combination of mathematical and narrative sub-group analyses to explore whether the results were homogenous when subgroups of studies were compared. Accordingly results were compared for men versus women, for self-reported versus clinically rated depression/depressive symptoms, for general population studies versus specific occupational cohorts and for high/medium high quality studies. Inspection of the confidence interval distributions, as well as sub-populations' summary odds ratios and their confidence intervals showed homogenous results across those dichotomies.

A limitation of our review could be that we may have underestimated the importance of work environment factors that have not been subjected to many empirical studies. There were examples of exposure factors that were examined in many studies but did not achieve level 3 in the grading system such as psychological demands - which only yielded evidence level 2. This illustrates the need for more detailed studies of different aspects of demands, such as emotional demands. In addition, effort reward imbalance was consistently associated with worsening depressive symptoms in three studies of medium high quality and was classified as second grade evidence. The summarized odds ratio was 1.78 which is comparable to the corresponding odds ratio for job strain which was 1.74. However, job strain had been examined in 14 studies and therefore achieved third grade evidence.

Another limitation is that we have not included outside work factors that may be of importance. As pointed out for instance by Wang and Schmitz [26] job strain may interact with psychosocial factors outside of the workplace in relation to the risk of major depression, and such interactions may in addition differ between men and women.

### Societal relevance

Despite the often moderate sizes of our findings, some of the associations are of considerable societal importance. An illustration of this is that if a work environment factor has a prevalence of 25 % and is associated with a relative risk of 1.8, the resulting population attributable risk is 11 %. Accordingly, when an exposure is common (as is the case with job strain, low level of control and effort reward imbalance with the operational definitions that have been used) even a moderately elevated risk associated with it becomes important in a societal context.

The work environment factors for which we found scientific evidence for an association to depressive symptom development are possible to influence by means of work organization changes. For instance, it has been shown that decision latitude for employees can be improved by analysis of the work organization with subsequent goal-directed organization intervention [27, 28] or by a year-long education of managers about psychosocial factors [29]. A review of natural experiments designed to reduce psychosocial risks in the work environment for bus drivers showed that such interventions may result in reduced biological stress in that group [30]. The present results suggest that in assessment and treatment plans of depression, work environment should be taken into account.

## Conclusions

There is substantial empirical evidence that employees, both men and women, who report lack of decision latitude, job strain and bullying, will experience increasing depressive symptoms over time.

Many of the work environmental factors can be favorably influenced by effective organizational interventions. An important step in this research field would be the launching of good evaluations of psychosocial interventions. For some kinds of working conditions that are developing in the modern working world, new research on depressive symptoms will be needed.

## Appendix

Studies judged to be of high or medium high quality

Ahola K, Hakanen J. Job strain, burnout, and depressive symptoms: a prospective study among dentists. J Affect Disord 2007;104:103-10.

Andrea H, Bultmann U, van Amelsvoort LG, Kant Y. The incidence of anxiety and depression among employees – the role of psychosocial work characteristics. Depress Anxiety 2009;26:1040-8.

Beseler CL, Stallones L, Hoppin JA, Alavanja MC, Blair A, Keefe T, et al. Depression and pesticide exposures among private pesticide applicators enrolled in the Agricultural Health Study. Environ Health Perspect 2008;116:1713-9.

Bonde JP, Munch-Hansen T, WieclawJ, Westergaard-Nielsen N, Agerbo E. Psychosocial work environment and antidepressant medication: a prospective cohort study. BMC Public Health 2009;9:262.

Burgard SA, Brand JE, House JS. Perceived job insecurity and worker health in the United States. Soc Sci Med 2009;69:777-85.

Clays E, De Bacquer D, Leynen F, Kornitzer M, Kittel F, De Backer G. Job stress and depression symptoms in middle-aged workers – prospective results from the Belstress study. Scand J Work Environ Health 2007;33:252-9.

Clumeck N, Kempenaers C, Godin I, Dramaix M, Kornitzer M, Linkowski P, et al. Working conditions predict incidence of long-term spells of sick leave due to depression: results from the Belstress I prospective study. J Epidemiol Community Health 2009;63:286-9

Dagher RK, McGovern PM, Dowd BE, Lundberg U. Postpartum depressive symptoms and the combined load of paid and unpaid work: a longitudinal analysis. Int Arch Occup Environ Health 2011;84:735-43.

De Lange AH, Taris TW, Kompier MAJ, Houtman ILD, Bongers PM. The relationships between work characteristics and mental health: examining normal, reversed and reciprocal relationships in a 4-wavestudy. Work & Stress 2004;18:149-66.

de Lange AH, Taris TW, Kompier MA, Houtman IL, Bongers PM. Effects of stable and changing demand-control histories on worker health. Scand J Work Environ Health 2002;28:94-108.

d’Errico A, Cardano M, LandriscinaT, Marinacci C, Pasian S, Petrelli A, et al. Workplace stress and prescription of antidepressant medications: a prospective study on a sample of Italian workers. Int Arch OccupEnviron Health 2011;84:413-24.

De Santo Iennaco J, Cullen MR, Cantley L, Slade MD, Fiellin M, Kasl SV. Effects of externally rated job demand and control on depression diagnosis claims in an industrial cohort. Am J Epidemiol 2010;171:303-11.

Fandino-Losada A, Forsell Y, Lundberg I. Demands, skill discretion, decision authority and social climate at work as determinants of major depression in a 3-year follow-up study. Int Arch Occup Environ Health 2013;86:591-605. Epub 2012 Jul 4.

Godin I, Kittel F, Coppieters Y, Siegrist J. A prospective study of cumulative job stress in relation to mental health. BMC Public Health 2005;5:67.

Goodman WB, Crouter AC. Longitudinal associations between maternal work stress, negative work-family spillover, and depressive symptoms. Fam Relat 2009;58:245-58.

Griffin JM, Fuhrer R, Stansfeld SA, Marmot M. The importance of low control at work and home on depression and anxiety: do these effects vary by gender and social class? Soc Sci Med 2002;54:783-98.

Grynderup MB, Mors O, Hansen AM, Andersen JH, Bonde JP, Kaergaard A, et al. Work-unit measures of organisational justice and risk of depression –a 2-year cohort study. Occup Environ Med 2013;70:380-5.

Grynderup MB, Mors O, Hansen AM, Andersen JH, Bonde JP, Kaergaard A, et al. A two-year follow-up study of risk of depression according to work-unit measures of psychological demands and decision latitude. Scand J Work Environ Health 2012;38:527-36.

Grzywacz JG, Quandt SA, Chen H, Isom S, Kiang L, Vallejos Q, et al. Depressive symptoms among Latino farm workers across the agricultural season: structural and situational influences. Cultur Divers Ethnic Minor Psychol 2010;16:335-43.

Ibrahim S, Smith P, Muntaner C. A multi-group cross-lagged analysis of work stressors and health using a Canadian National sample. Soc Sci Med 2009;68:49-59.

Jensen HK, Wieclaw J, Munch-Hansen T, Thulstrup AM, Bonde JP. Does dissatisfaction with psychosocial work climate predict depressive, anxiety and substance abuse disorders? A prospective study of Danish public service employees. J Epidemiol Community Health 2010;64:796-801.

Kivimaki M, Vahtera J, Elovainio M, Virtanen M, Siegrist J. Effort-reward imbalance, procedural injustice and relational injustice as psychosocial predictors of health: complementary or redundant models? Occup Environ Med 2007;64:659-65.

Kivimäki M, Virtanen M, Vartia M, Elovainio M, Vahtera J, Keltikangas-Jarvinen L. Workplace bullying and the risk of cardiovascular disease and depression. Occup Environ Med 2003;60:779- 83.

Kouvonen A, Oksanen T, Vahtera J, Stafford M, Wilkinson R, Schneider J, et al. Low workplace social capital as a predictor of depression: the Finnish Public Sector Study. Am J Epidemiol 2008;167:1143-51.

Lang J, Bliese PD, Lang JW, Adler AB. Work gets unfair for the depressed: cross-lagged relations between organizational justice perceptions and depressive symptoms. J Appl Psychol 2011;96:602-18.

Levin A, Besser A, Albert L, Smith D, Neria Y. The effect of attorneys’ work with trauma- exposed clients on PTSD symptoms, depression, and functional impairment: a cross-lagged longitudinal study. Law Hum Behav 2012;36:538-47.

Magnusson Hanson LL, Theorell T, Bech P, Rugulies R, Burr H, Hyde M, et al. Psychosocial working conditions and depressive symptoms among Swedish employees. Int Arch Occup Environ Health 2009;82:951-60.

Mantyniemi A, Oksanen T, Salo P, Virtanen M, Sjosten N, Pentti J, et al. Job strain and the risk of disability pension due to musculoskeletal disorders, depression or coronary heart disease: a prospective cohort study of 69,842 employees. Occup Environ Med 2012;69:574-81.

Niedhammer I, Goldberg M, Leclerc A, Bugel I, David S. Psychosocial factors at work and subsequent depressive symptoms in the Gazel cohort. Scand J Work Environ Health 1998;24:197-205.

Oksanen T, Kouvonen A, Vahtera J, Virtanen M, Kivimäki M. Prospective study of workplace social capital and depression: are vertical and horizontal components equally important? J Epidemiol Community Health 2010;64:684-9.

Parker SK. Longitudinal effects of lean production on employee outcomes and the mediating role of work characteristics. J Appl Psychol 2003;88:620-34.

Paterniti S, Niedhammer I, Lang T, Consoli SM. Psychosocial factors at work, personality traits and depressive symptoms. Longitudinal results from the GAZEL Study. Br J Psychiatry 2002;181:111-7.

Plaisier I, de Bruijn JG, de Graaf R, ten Have M, Beekman AT, Penninx BW. The contribution of working conditions and social support to the onset of depressive and anxiety disorders among male and female employees. Soc Sci Med 2007;64:401-10.

Rugulies R, Aust B, Madsen IE, Burr H, Siegrist J, Bultmann U. Adverse psychosocial working conditions and risk of severe depressive symptoms. Do effects differ by occupational grade? Eur J Public Health 2013;23:415-20. Epub 2012 Jun 8.

Rugulies R, Madsen IE, Hjarsbech PU, Hogh A, Borg V, Carneiro IG, et al. Bullying at work and onset of a major depressive episode among Danish female eldercare workers. Scand J Work Environ Health 2012;38:218-27.

Rugulies R, Thielen K, Nygaard E, Diderichsen F. Job insecurity and the use of antidepressant medication among Danish employees with and without a history of prolonged unemployment: a 3.5-year follow-up study. J Epidemiol Community Health 2010;64:75-81.

Rugulies R, Bultmann U, Aust B, Burr H. Psychosocial work environment and incidence of severe depressive symptoms: prospective findings from a 5-yearfollow-up of the Danish work environment cohort study. Am J Epidemiol 2006;163:877-87.

Schonfeld IS. Stress in 1st-year women teachers: the context of social support and coping. Genet Soc Gen Psychol Monogr 2001;127:133-68.

Shields M. Stress and depression in the employed population. Health Rep 2006;17:11-29.

Shields M. Long working hours and health. Health Rep 1999;11:33-48 (Eng); 37-55 (Fre).

Sinokki M, Hinkka K, Ahola K, Koskinen S, Kivimäki M, Honkonen T, et al. The association of social support at work and in private life with mental health and antidepressant use: the Health 2000 Study. J Affect Disord 2009;115:36-45.

Stansfeld SA, Shipley MJ, Head J, Fuhrer R. Repeated job strain and the risk of depression: longitudinal analyses from the Whitehall II study. Am J Public Health 2012;102:2360-6.

Stoetzer U, Ahlberg G, Johansson G, Bergman P, Hallsten L, Forsell Y, et al. Problematic interpersonal relationships at work and depression: a Swedish prospective cohort study. J Occup Health 2009;51:144-51.

Theorell T, Nyberg A, Leineweber C, Magnusson Hanson LL, Oxenstierna G, Westerlund H. Non-listening and self centered leadership – relationships to socioeconomic conditions and employee mental health. PLoS One 2012;7:e44119.

Wang J, Schmitz N. Does job strain interact with psychosocial factors outside of the workplace in relation to the risk of major depression? The CanadianNational Population Health Survey. Soc Psychiatry Psychiatr Epidemiol 2011;46:577-84.

Wang J, Schmitz N, Dewa C, Stansfeld S. Changes in perceived job strain and the risk of major depression: results from a population-based longitudinal study. Am J Epidemiol 2009;169:1085- 91.

Wang J. Work stress as a risk factor for major depressive episode(s). Psychol Med 2005;35:865- 71.

Wang J. Perceived work stress and major depressive episodes in a population of employed Canadians over 18 years old. J Nerv Ment Dis 2004;192:160-3.

Varma A, Marott JL, Stoltenberg CD, Wieclaw J, Kolstad HA, Bonde JP. With long hours of work, might depression then lurk? A nationwide prospective follow-up study among Danish senior medical consultants. Scand J Work Environ Health 2012;38:418-26.

Weisskopf MG, Moisan F, TzourioC, Rathouz PJ, Elbaz A. Pesticide exposure and depression among agricultural workers in France. Am J Epidemiol 2013;178:1051-8.

Wieclaw J, Agerbo E, Mortensen PB, Burr H, Tuchsen F, Bonde JP. Psychosocial working conditions and the risk of depression and anxiety disorders in the Danish workforce. BMC Public Health 2008;8:280.

Wieclaw J, Agerbo E, Mortensen PB, Burr H, Tuchsen F, Bonde JP. Work related violence and threats and the risk of depression and stress disorders. J Epidemiol Community Health 2006;60:771-5.

Virtanen M, Stansfeld SA, Fuhrer R, Ferrie JE, Kivimäki M. Overtime work as a predictor of major depressive episode: a 5-year follow-up of the Whitehall II study. PLoS One 2012;7:e30719.

Virtanen M, Ferrie JE, Singh-Manoux A, Shipley MJ, Stansfeld SA, Marmot MG, et al. Long working hours and symptoms of anxiety and depression: a 5-year follow-up of the Whitehall II study. Psychol Med 2011;41:2485-94.

Virtanen M, Batty GD, Pentti J, Vahtera J, Oksanen T, Tuisku K, et al. Patient overcrowding in hospital wards as a predictor of diagnosis-specific mental disorders among staff: a 2-year prospective cohort study. J ClinPsychiatry 2010;71:1308-12.

Virtanen M, Honkonen T, KivimäkiM, Ahola K, Vahtera J, Aromaa A, et al. Work stress, mental health and antidepressant medication findings from the Health 2000 Study. J Affect Disord 2007;98:189-97.

Ybema JF, van den Bos K. Effects of organizational justice on depressive symptoms and sickness absence: a longitudinal perspective. Soc Sci Med 2010;70:1609-17.

Ylipaavalniemi J, Kivimaki M, Elovainio M, Virtanen M, Keltikangas-Jarvinen L, Vahtera J. Psychosocial work characteristics and incidence of newly diagnosed depression: a prospective cohort study of three different models. Soc Sci Med 2005;61:111-22.
